# Association between Preoperative Glucose Dysregulation and Delirium after Non-Cardiac Surgery

**DOI:** 10.3390/jcm13040932

**Published:** 2024-02-06

**Authors:** Ah Ran Oh, Dong Yun Lee, Seunghwa Lee, Jong-Hwan Lee, Kwangmo Yang, Byungjin Choi, Jungchan Park

**Affiliations:** 1 Department of Anesthesiology and Pain Medicine, Samsung Medical Center, Sungkyunkwan University School of Medicine, Seoul 06351, Republic of Korea; 2 Department of Anesthesiology and Pain Medicine, Kangwon National University Hospital, Chuncheon 24289, Republic of Korea; 3 Department of Biomedical Informatics, Ajou University Graduate School of Medicine, Suwon 16499, Republic of Korea; 4 Rehabilitation & Prevention Center, Heart Vascular Stroke Institute, Samsung Medical Center, Sungkyunkwan University School of Medicine, Seoul 06351, Republic of Korea; 5 Department of Biomedical Engineering, Seoul National University College of Medicine, Seoul 03080, Republic of Korea; 6 Center for Health Promotion, Samsung Medical Center, Sungkyunkwan University School of Medicine, Seoul 06351, Republic of Korea

**Keywords:** delirium, glucose, non-cardiac surgery

## Abstract

This study aimed to investigate the association between glucose dysregulation and delirium after non-cardiac surgery. Among a total of 203,787 patients who underwent non-cardiac surgery between January 2011 and June 2019 at our institution, we selected 61,805 with available preoperative blood glucose levels within 24 h before surgery. Patients experiencing glucose dysregulation were divided into three groups: hyperglycemia, hypoglycemia, and both. We compared the incidence of postoperative delirium within 30 days after surgery between exposed and unexposed patients according to the type of glucose dysregulation. The overall incidence of hyperglycemia, hypoglycemia, and both was 5851 (9.5%), 1452 (2.3%), and 145 (0.2%), respectively. The rate of delirium per 100 person-months of the exposed group was higher than that of the unexposed group in all types of glucose dysregulation. After adjustment, the hazard ratios of glucose dysregulation in the development of delirium were 1.35 (95% CI, 1.18–1.56) in hyperglycemia, 1.36 (95% CI, 1.06–1.75) in hypoglycemia, and 3.14 (95% CI, 1.27–7.77) in both. The subgroup analysis showed that exposure to hypoglycemia or both to hypo- and hyperglycemia was not associated with delirium in diabetic patients, but hyperglycemia was consistently associated with postoperative delirium regardless of the presence of diabetes. Preoperative glucose dysregulation was associated with increased risk of delirium after non-cardiac surgery. Our findings may be helpful for preventing postoperative delirium, and further investigations are required to verify the association and mechanisms for the effect we observed.

## 1. Introduction

Delirium is a commonly found complication after surgery, characterized by fluctuating disturbances in cognition or attention during the postoperative period [1]. Although most cases spontaneously resolve, delirium is related to increased durations of hospital stay, healthcare costs, complications, readmission rates, and even in-hospital mortality [2,3,4,5]. The exact mechanism causing postoperative delirium is not clear. Any condition related to cerebral vulnerability or exogenous neurocognitive stress may be involved, and the overlap between predisposing and precipitating factors makes it more difficult to predict and prevent postoperative delirium. Multi-component interventions have been proposed to prevent postoperative delirium in frail patients, but guidelines have not been established [6].

Glycemic control is a cornerstone in perioperative care. Hypoglycemia may be directly related to delirious status, and theoretically, hyperglycemia may also cause neuronal damage by inducing oxidative stress, which compromises the blood–brain barrier [7]. In fact, glucose dysregulation in diabetic patients has been shown to increase the incidence of delirium in patients treated in intensive care units [8], but this association has not been thoroughly investigated in surgical patients. Surgical patients frequently encounter risks from both hypoglycemia from preoperative fasting and hyperglycemia from surgical stress. Moreover, glucose dysregulation during the perioperative period is commonly reported even in non-diabetic patients [9]. For this reason, the present study retrospectively analyzed the association between preoperative blood glucose level within 24 h measured before non-cardiac surgery and the development of postoperative delirium.

## 2. Methods

The Institutional Review Board of Samsung Medical Center approved this study (Samsung Medical Center, 81 Irwon-ro, Gangnam-gu, Seoul, Republic of Korea, 2021-06-078-001 Chairperson Prof. SW Park, date of approval 5 July 2021). It was conducted following the Declaration of Helsinki. The need for informed consent from individual patients was waived because the registry for this study was initially curated in de-identified form. Our script followed the Strengthening the Reporting of Observational Studies in Epidemiology reporting guidelines.

### 2.1. Study Population and Data Sources

This study used data from the Samsung Medical Center—Non-Cardiac operation (SMC-NoCop) registry (KCT 0006363). This cohort contains single-center de-identified data of 203,787 consecutive adult patients who underwent non-cardiac surgery at Samsung Medical Center, Seoul, Republic of Korea, between January 2011 and June 2019. For the analysis, we excluded patients who had a preoperative history of delirium or dementia and those without blood glucose level measurements within 24 h before surgical incision.

The raw data for this registry were extracted using Clinical Data Warehouse Darwin-C from Samsung Medical Center, which is an electronic system built to enable investigators to search and retrieve medical records from the institutional electronic archive system in de-identified form. The hospital records of more than 4 million patients, comprising more than 900 million laboratory findings and 200 million prescriptions, are available in this system. It also contains mortality data drawn from outside the institution using the National Population Registry of the Korea National Statistical Office, in which each record has a unique personal identification number.

### 2.2. Exposure

The exposure for this study was glucose dysregulation including hyperglycemia, hypoglycemia, and both. Hyperglycemia was defined as at least one fasting blood glucose level >140 mg/dL or random glucose >180 mg/dL within 24 h before surgical incision according to the American Diabetes Association and American Association of Clinical Endocrinology guidelines [10]. Hypoglycemia was defined as at least one measured glucose level being lower than 70 mg/dL within 24 h before surgical incision. We also investigated the risk of delirium for patients with both hyper- and hypoglycemia within 24 h before surgery. According to our institutional protocol, preoperative blood glucose level was selectively measured for those with a remarkable medical history, diabetes, or higher surgical risk.

### 2.3. Outcome

The primary endpoint was postoperative delirium within 30 days after the operation, and the secondary endpoints were mortalities during one- and three-year follow-ups. Patients with acute confusion or behavioral changes during the postoperative period were referred to the Department of Psychiatry at the discretion of attending clinicians, and postoperative delirium was diagnosed by psychiatrists using the confusion assessment method.

### 2.4. Covariates

Investigators who were independent from this study extracted raw data and organized relevant variables including demographic data, underlying diseases, and information from blood laboratory tests. Underlying diseases were defined based on the International Classification of Diseases-10 codes and included not only medical comorbidities such as hypertension, diabetes, and stroke, but also psychiatric disorders such as mood and substance use disorders. The surgical risk was stratified into low, intermediate, and high groups following the Society of Cardiology/European Society of Anaesthesiology guidelines on non-cardiac surgery [11].

### 2.5. Statistical Analysis

The baseline characteristics of patients are presented and compared according to presence of postoperative delirium. Continuous variables are presented as mean ± standard deviation or median with interquartile range, and comparisons were conducted using a *t*-test or Mann–Whitney test, as applicable. Categorical variables are presented as numbers and percentages and were compared using a Chi-square or Fisher’s exact test. We also described the distributions of baseline characteristics among patients who were and were not exposed to glucose dysregulation and applied inverse probability of weighting (IPW) by computing stabilized weights inversely proportional to cohort members’ probability of glucose dysregulation exposure [12]. We used standardized differences to assess balance between two groups and considered an absolute value of less than 0.10 as negligible. Patients with missing data were excluded from this study. A subgroup analysis was conducted in patients without a preoperative psychiatric disorder (Supplementary Table S1). We used Kaplan–Meier analysis with the log-rank test to compare the cumulative incidence of delirium between exposed and unexposed patients according to the type of glucose dysregulation. Next, we used Cox proportional hazard regression to generate hazard ratios (HR) with 95% confidence intervals (CI) for delirium by comparing exposed and unexposed patients, adjusting for potential confounding factors using IPW. The same analysis method including Kaplan–Meier analysis and Cox proportional hazard regression was applied to the secondary outcome of mortality. We also conducted subgroup analyses using the same analytic method comparing individuals with and without diabetes. All statistical analyses were performed using R 4.2.0 (Vienna, Austria; http://www.R-project.org/ (accessed on 3 February 2023)).

## 3. Results

**Figure 1:**
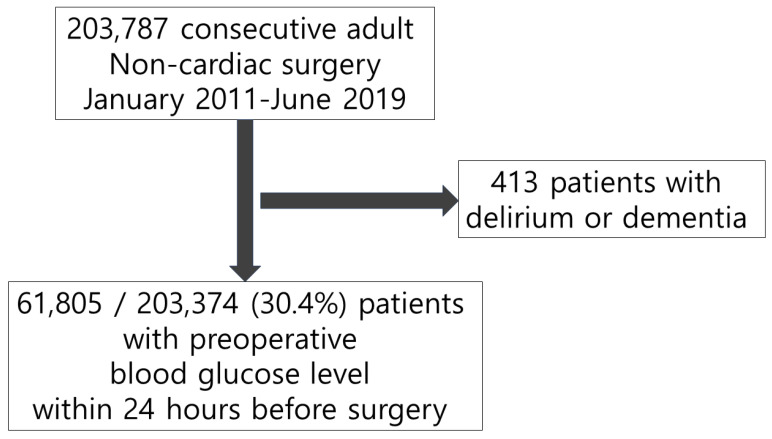
Flowchart of study population.

We excluded 413 patients with preoperative diagnoses of delirium or dementia and enrolled 61,805 of 203,374 (30.4%) patients with available preoperative blood glucose level within 24 h before surgical incision (Figure 1). From a total of 61,805 study patients, 26,985 patients underwent psychiatric evaluation owing to acute confusion or behavioral changes during the postoperative period. Delirium was diagnosed in 1936 (3.1%) patients. The baseline characteristics of patients with and without delirium are summarized in Table 1. During the 24 h before surgical incision, hyperglycemia was observed in 5851 (9.5%) patients, and its incidence was 9.1% in patients without delirium and 20.4% in those with delirium. The overall incidence of hypoglycemia was 2.3% (1452/61,805), with 2.3% in the no-delirium and 4.4% in the delirium groups. The patients in the delirium group were older, more likely to be male, and showed a higher incidence of comorbidities (Table 1). The baseline characteristics are summarized according to the presence of glycemic dysregulation (Supplementary Tables S2–S4). The balance of covariates between groups before and after IPW adjustment are presented in ASD.

**Figure 2:**
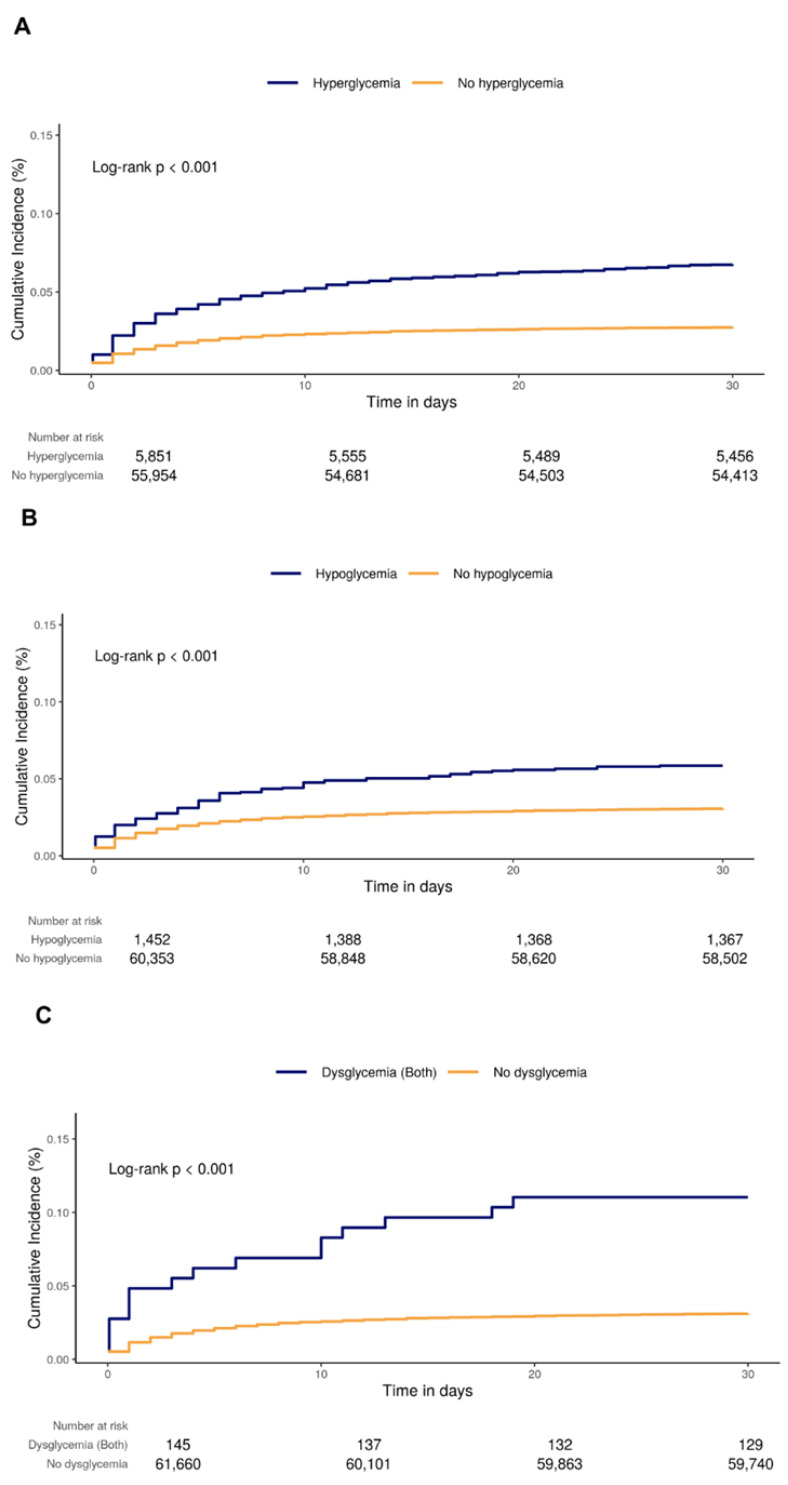
Kaplan–Meier curves of delirium according to exposure to (**A**) hyperglycemia, (**B**) hypoglycemia, and (**C**) both.

The risks of postoperative delirium are presented in Table 2. The rate per 100 person-months of the exposed group was higher than that of the unexposed group for all types of glucose dysregulation (hyperglycemia: exposed 7.13 [95% CI, 6.45–7.87] vs. unexposed 2.82 [95% CI, 2.68–2.96]; hypoglycemia: exposed 6.14 [95% CI, 4.91–7.59] vs. unexposed 3.15 [95% CI, 3.01–3.29]; both: exposed 12.12 [95% CI, 6.93–19.68] vs. unexposed 3.20 [95% CI, 3.05–3.34], respectively). A subgroup analysis in patients without psychiatric disorder showed consistent results (in the Kaplan–Meier analysis, the cumulative incidences of the exposed groups were higher than those of the unexposed groups for all types of glucose dysregulation (hyperglycemia: log-rank *p* < 0.001; hypoglycemia: log-rank *p* < 0.001; both: log-rank *p* < 0.001)) (Figure 2). In the primary analysis for postoperative delirium using a Cox proportional hazards model with IPW, the adjusted HRs were 1.35 (95% CI, 1.18–1.56) in hyperglycemia, 1.36 (95% CI, 1.06–1.75) in hypoglycemia, and 3.14 (95% CI, 1.27–7.77) in both (Table 2). In subgroup analyses, the trends of the incidence rate did not substantively change compared with the primary analysis. However, there was a difference from the primary analysis in the Cox proportional hazards model. In patients with diabetes, exposure to hyperglycemia alone was significant, without HR overlap (adjusted HR, 1.33; 95% CI, 1.10–1.62). In the group of patients without diabetes, exposure to hyperglycemia and exposure to hypoglycemia did not overlap (hyperglycemia: adjusted HR, 1.32 [95% CI, 1.06–1.65]; hypoglycemia: adjusted HR, 1.77 [95% CI, 1.21–2.59], respectively).

The risks of mortality are presented in Table 3. The rate per 100 person-months of the exposed group was higher than that of the unexposed group for all types of glucose dysregulation. For hyperglycemia, the one-year mortality was 1.07 (95% CI, 0.99–1.15) in the exposed group and 0.41 (95% CI, 0.40–0.43) in the unexposed group. In the Kaplan–Meier analysis, the cumulative incidence of three-year mortality of the exposed group was higher than that of the unexposed group for all types of glucose dysregulation except exposure to hypoglycemia (Figures S1 and S2). In the secondary analysis of mortality using a the Cox proportional hazards model with IPW, the adjusted HRs of one-year and three-year mortalities were 1.32 (95% CI, 1.18–1.47) and 1.32 (95% CI, 1.21–1.44) in hyperglycemia, 1.03 (95% CI, 0.73–1.28) and 0.88 (95% CI, 0.73–1.05) in hypoglycemia, and 1.22 (95% CI, 0.52–2.84) and 3.89 (95% CI, 1.02–14.73) in both (Table 3), respectively. In the subgroup analyses, trends of the incidence rate did not substantively change compared to the secondary analysis. There were similar trends in the secondary analysis using a Cox proportional hazards model. Specifically, only exposure to hyperglycemia showed consistently significant results (one-year mortality with diabetes: adjusted HR, 1.37 [95% CI, 1.17–1.59]; one-year mortality without diabetes: adjusted HR, 1.32 [95% CI, 1.09–1.60]; three-year mortality with diabetes: adjusted HR, 1.40 [95% CI, 1.25–1.56]; three-year mortality without diabetes: adjusted HR, 1.24 [95% CI, 1.07–1.45]) (Table 3).

## 4. Discussion

In our population-based cohort study, exposure to hyperglycemia within 24 h before non-cardiac surgery was associated with a 35% relative increase in the risk of postoperative delirium within 30 days after surgery compared with those unexposed after IPW adjustment. Patients who were exposed to hyperglycemia were also significantly associated with an increased risk of one-year and three-year mortalities compared to patients who were unexposed.

Exposure to preoperative hyperglycemia was consistently associated with postoperative delirium regardless of the presence of diabetes. This is in line with the results of previous population-based retrospective cohort studies that found an association between elevated preoperative fasting glucose level and an increased risk of postoperative delirium [13,14]. Several possible explanations for the effects of hyperglycemia on postoperative delirium include surgical stress that downregulates parasympathetic tone and upregulates sympathetic tone, the release of neuroinflammation, damage to brain oxidative metabolism, and abnormal neurotransmitter pathways [15]. It has also been reported that hyperglycemia could directly induce neuroinflammation [16]. Specifically, blood–brain barrier permeability can be changed by hyperglycemia and result in neuroinflammation, oxidative damage, and cognitive dysfunction [7]. Another possible explanation is that in-hospital patients are likely to experience hyperglycemia related to acute stress, and stress hyperglycemia is associated with an increased risk of delirium [17].

Contrary to the effects of exposure to hyperglycemia, our results for hypoglycemia or both hyperglycemia and hypoglycemia showed inconsistent relationships with postoperative delirium. A delirious state associated with hypoglycemia has long been reported [18,19,20,21]. It can be explained by neurocognitive performance alterations owing to hypoglycemia, but hypoglycemia may account only partially for patients with delirium [17,22], In fact, the effect of hypoglycemia on delirium remains controversial. A previous study of patients in the intensive care unit showed that hyperglycemia was associated with delirium [8], but hypoglycemia was not. In diabetic patients, there is a recent report stating that relative hypoglycemia falling below the normal blood glucose target is associated with delirium in critically ill patients rather than hypoglycemia defined by an absolute blood glucose level [23]. In the present study, there were only about 100 patients with both hyperglycemia and hypoglycemia, accounting for 0.2% of the total cohort, so the effect on both hyperglycemia and hypoglycemia may be underestimated. Therefore, the effect of both hyperglycemia and hypoglycemia on postoperative delirium should be explored in further research with a different threshold of hypoglycemia and a sufficient number of patients.

Preoperative hyperglycemia was also associated with increased postoperative one-year and three-year mortalities regardless of the patient’s history of diabetes. Several studies have shown that preoperative hyperglycemia increases short- and long-term mortalities after non-cardiac surgery [9,24,25], but the underlying mechanisms are not completely understood. A possible pathophysiological mechanism for this finding may lie in the increase in pro-inflammatory mediators and reactive oxygen species induced by hyperglycemia, causing immune dysfunction and direct cellular damage [26]. These responses may be a chronic process that affects long-term mortality and appear to influence diabetic patients to a similar degree to those without diabetes. However, in this study, the effect of hyperglycemia in patients with diabetes differed from prior studies [9,25], which may be attributable to the abnormal mixed glucose dysregulation of our control group. Therefore, further investigations to verify the association between hyperglycemia and mortality in diabetic patients undergoing non-cardiac surgery are warranted.

Another intriguing finding of our study was that patients exposed to both hyperglycemia and hypoglycemia had three-fold increased three-year mortality compared to those with only hyperglycemia. This result may be biologically explained by the fact that markers of oxidative stress were maintained at higher levels when the glucose level was altered than when hyperglycemia was sustained [27]. Notably, the adverse impact of glucose fluctuation was significant only in patients without diabetes. We hypothesize that patients with diabetes developed relative tolerance to the cellular and microvascular complications associated with glucose fluctuation [28]. This finding implies that minimizing glucose fluctuations is important regardless of the presence of diabetes.

This study has several limitations. First, due to its retrospective nature, unmeasured confounding factors may have affected our results despite the use of rigorous statistical adjustments. In particular, the lack of consideration for intraoperative and postoperative glucose levels may have played a role in the development of postoperative outcomes. Moreover, the observed prevalence of delirium in our study was lower compared to the rates reported in previous studies. This discrepancy may be attributed, at least in part, to the absence of systematic screening for delirium in the postoperative period. Several studies have demonstrated that when not routinely assessing for delirium, over 50% of cases can go undetected. In our real-world setting, the absence of systematic screening may have led to an underestimation of delirium prevalence, introducing a significant source of uncertainty into the outcome measure of this study. Despite the low observed prevalence, it is essential to note that this study included a substantial number of patients, surpassing the participant numbers in some comparable studies. While this large sample size can provide valuable insights, it may also contribute to the inevitably low prevalence of outcomes, such as delirium. Correct measurement and interpretation of the results are crucial, and the lack of systematic screening for delirium postoperatively should be considered when interpreting the findings. Second, the measurement of preoperative blood glucose levels was conducted in accordance with institutional protocols, but was also subject to clinician discretion, particularly in patients without a known history of diabetes. This discretionary aspect may have caused selection bias, as the decision to measure blood glucose could have been influenced by the clinical condition or perceived risk of certain patients. Consequently, our observations may primarily apply to those individuals for whom clinicians deemed glucose measurement necessary, potentially limiting the generalizability of our findings to a broader noncardiac surgery population. Additionally, we only included patients with preoperative glucose measurements taken within 24 h before surgery. While this timeframe was chosen to capture a relevant preoperative status, it may have inadvertently resulted in a selection bias. Patients with glucose measurements might represent a subset with specific characteristics or indications for testing, introducing potential confounding factors not fully accounted for in our analysis. Third, we could not address whether glucose control during the preoperative period reduced postoperative delirium or mortality. In particular, strict glucose control has been associated with adverse outcomes in other clinical settings [29,30,31], so it is unclear whether intensified regulation will improve clinical outcomes in non-cardiac surgery. Despite these limitations, preoperative glucose dysregulation was associated with increased risk of delirium. This result suggests that preoperative glucose level should be considered when predicting postoperative delirium and be treated during the perioperative period in non-cardiac surgical patients.

## 5. Conclusions

Preoperative glucose dysregulation was associated with increased postoperative delirium after non-cardiac surgery. In diabetic patients, exposure to hypoglycemia or both to hypo- and hyperglycemia was not associated with delirium in diabetic patients, while hyperglycemia was consistently associated with postoperative delirium regardless of the presence of diabetes. Our findings may be helpful for preventing postoperative delirium, and further investigations are required to verify the association and mechanisms for the effect we observed. Further prospective studies should be conducted to verify this association and evaluate potential mechanisms in non-cardiac surgery.

## Figures and Tables

**Table 1:** Baseline characteristics and outcomes of patients with and without delirium.

	No Delirium (*n* = 59,869)	Delirium (*n* = 1936)	*p* Value
Hyperglycemia	5456 (9.1)	395 (20.4)	<0.001
Hypoglycemia	1367 (2.3)	85 (4.4)	<0.001
Glycemic dysregulation	129 (0.2)	16 (0.8)	<0.001
Male	31,508 (52.6)	1297 (67.0)	<0.001
Age	57.5 (±14.6)	64.7 (±14.6)	<0.001
Body mass index	24.4 (±3.7)	23.6 (±3.8)	<0.001
Psychiatric disorder, any	2628 (4.4)	243 (12.6)	<0.001
Mood disorder	1133 (1.9)	114 (5.9)	<0.001
Schizophrenia	63 (0.1)	21 (1.1)	<0.001
Alcoholic use disorder	65 (0.1)	10 (0.5)	<0.001
Other substance abuse	15 (0.0)	3 (0.2)	0.01
Sleep disorder	893 (1.5)	56 (2.9)	<0.001
Personality disorder	20 (0.0)	2 (0.1)	0.32
Current alcohol	11,119 (18.6)	288 (14.9)	<0.001
Current smoking	5160 (8.6)	229 (11.8)	<0.001
Previous disease			
Hypertension	23,009 (38.4)	912 (47.1)	<0.001
Diabetes	20,908 (34.9)	677 (35.0)	0.99
Chronic kidney disease	2208 (3.7)	155 (8.0)	<0.001
Dialysis	702 (1.2)	93 (4.8)	<0.001
Stroke	2262 (3.8)	172 (8.9)	<0.001
Coronary artery disease	2144 (3.6)	124 (6.4)	<0.001
Heart failure	404 (0.7)	35 (1.8)	<0.001
Arrhythmia	1470 (2.5)	118 (6.1)	<0.001
Peripheral artery disease	367 (0.6)	35 (1.8)	<0.001
Aortic disease	357 (0.6)	43 (2.2)	<0.001
Valvular heart disease	134 (0.2)	11 (0.6)	<0.001
Chronic obstructive pulmonary disease	1459 (2.4)	98 (5.1)	<0.001
Preoperative blood laboratory tests			
Hemoglobin, g/dL	13.1 (±1.9)	11.9 (±2.3)	<0.001
Creatinine, mg/dL	1.0 (±1.3)	1.4 (±2.0)	<0.001
Preoperative electrolytes			
Hyponatremia	3323 (5.6)	375 (19.4)	<0.001
Hypernatremia	594 (1.0)	25 (1.3)	0.24
Hypokalemia	1308 (2.2)	127 (6.6)	<0.001
Hyperkalemia	787 (1.3)	61 (3.2)	<0.001
Hypophosphatemia	2010 (3.4)	168 (8.7)	<0.001
Hyperphosphatemia	2426 (4.1)	134 (6.9)	<0.001
Hypochloremia	2056 (3.4)	201 (10.4)	<0.001
Hyperchloremia	9906 (16.5)	493 (25.5)	<0.001
Operative variables			
General anesthesia	53,625 (89.6)	1795 (92.7)	<0.001
Emergency operation	8148 (13.6)	584 (30.2)	<0.001
Operation duration, min	165.6 (±121.0)	250.4 (±180.4)	<0.001
Intraoperative transfusion	4354 (7.3)	585 (30.2)	<0.001
Intraoperative inotropic infusion	8153 (13.6)	870 (44.9)	<0.001
Surgical risk			
Mild	12,713 (21.2)	218 (11.3)	<0.001
Intermediate	39,331 (65.7)	1161 (60.0)	<0.001
High	7825 (13.1)	557 (28.8)	<0.001
Surgery types			<0.001
Neuroendocrine	1320 (2.2)	5 (0.3)	
Lung	3179 (5.3)	113 (5.8)	
Head and neck	12,949 (21.6)	417 (21.5)	
Breast	1395 (2.3)	7 (0.4)	
Stomach	2881 (4.8)	52 (2.7)	
Hepatobiliary	8803 (14.7)	427 (22.1)	
Colorectal	4717 (7.9)	206 (10.6)	
Urology	6110 (10.2)	116 (6.0)	
Gynecology	3873 (6.5)	19 (1.0)	
Bone, skin, etc.	14,642 (24.5)	574 (29.6)	
Outcomes			
One-year mortality	3039 (5.1)	406 (21.0)	<0.001
Three-year mortality	5891 (9.8)	569 (29.4)	<0.001

Data are presented as *n* (%) or mean (±standard deviation). Surgical risk was stratified according to 2014 European Society of Cardiology/European Society of Anaesthesiology guidelines.

**Table 2:** Association between glucose dysregulation and delirium after non-cardiac surgery.

Outcome	Unexposed Group		Exposed Group			
	No. with Outcome/Total No. (%)	Incidence Rate per 100 Person-Months (95% CI)	No. with Outcome/Total No. (%)	Incidence Rate per 100 Person-Months (95% CI)	Crude HR (95% CI)	IPTW-Adjusted HR (95% CI)
Primary analysis						
Hyperglycemia	1541/55,954 (2.8)	2.82 (2.68–2.96)	395/5851 (6.8)	7.13 (6.45–7.87)	2.50 (2.24–2.80)	1.35 (1.18–1.56)
Hypoglycemia	1851/60,353 (3.1)	3.15 (3.01–3.29)	85/1452 (5.9)	6.14 (4.91–7.59)	2.50 (2.24–2.80)	1.36 (1.06–1.75)
Both	1920/61,660 (3.1)	3.20 (3.05–3.34)	16/145 (11.0)	12.12 (6.93–19.68)	3.72 (2.27–6.08)	3.14 (1.27–7.77)
Subgroup analysis						
With diabetes						
Hyperglycemia	482/18,452 (2.6)	2.67 (2.44–2.92)	195/3133 (6.2)	6.56 (5.67–7.55)	2.43 (2.06–2.87)	1.33 (1.10–1.62)
Hypoglycemia	637/20,759 (3.1)	3.15 (2.91–3.40)	40/826 (4.8)	5.05 (3.61–6.88)	1.59 (1.16–2.19)	1.25 (0.86–1.836)
Both	667/21,469 (3.1)	3.19 (2.95–3.44)	10/116 (8.6)	9.35 (4.48–17.2)	1.59 (1.16–2.19)	1.99 (0.52–7.65)
Without diabetes						
Hyperglycemia	1059/37,502 (2.8)	2.89 (2.72–3.07)	200/2718 (7.4)	7.80 (6.76–8.96)	2.67 (2.30–3.10)	1.32 (1.06–1.65)
Hypoglycemia	1214/39,594 (3.1)	3.14 (2.97–3.33)	45/626 (7.2)	7.61 (5.56–10.19)	2.39 (1.78–3.22)	1.77 (1.21–2.59)
Both	1253/40,191 (3.1)	3.20 (3.03–3.38)	6/29 (20.7)	25.00 (9.17–54.41)	7.37 (3.30–16.43)	1.22 (0.18–3.85)

HR = hazard ratio; CI = confidence interval.

**Table 3:** Association between glucose dysregulation and mortality after non-cardiac surgery.

Outcome	Unexposed Group		Exposed Group			
	No. with Outcome /Total No. (%)	Incidence Rate per 100 Person-Months (95% CI)	No. with Outcome /Total No. (%)	Incidence Rate per 100 Person-Months (95% CI)	Crude HR (95% CI)	IPTW-Adjusted HR (95% CI)
One-year follow-up						
Primary analysis						
Hyperglycemia	2747/55,954 (4.9)	0.41 (0.40–0.43)	698/5851 (11.9)	1.07 (0.99–1.15)	2.63 (2.42–2.85)	1.32 (1.18–1.47)
Hypoglycemia	3332/60,353 (5.5)	0.47 (0.45–0.48)	113/1452 (7.8)	0.68 (0.56–0.81)	1.43 (1.19–1.73)	1.03 (0.73–1.28)
Both	3426/61,660 (5.6)	0.47 (0.46–0.49)	19/145 (13.1)	1.20 (0.72–1.88)	2.48 (1.58–3.90)	1.22 (0.52–2.84)
Subgroup analysis						
With diabetes						
Hyperglycemia	805/18,452 (4.4)	0.37 (0.34–0.39)	316/3133 (10.1)	0.89 (0.79–0.99)	2.41 (2.12–2.75)	1.37 (1.17–1.59)
Hypoglycemia	1067/20,759 (5.1)	0.43 (0.41–0.46)	54/826 (6.5)	0.56 (0.42–0.73)	1.30 (0.99–1.70)	1.15 (0.84–1.56)
Both	1110/21,469 (5.2)	0.44 (0.41–0.46)	11/116 (9.5)	0.83 (0.42–1.49)	1.85 (1.02–3.35)	1.64 (0.60–4.51)
Without diabetes						
Hyperglycemia	1942/37,502 (5.2)	0.44 (0.42–0.46)	382/2718 (14.1)	1.30 (1.17–1.43)	3.09 (2.77–3.45)	1.32 (1.09–1.60)
Hypoglycemia	2265/39,594 (5.7)	0.49 (0.47–0.51)	59/626 (9.4)	0.84 (0.64–1.08)	1.69 (1.31–2.19)	0.99 (0.69–1.44)
Both	2316/40,191 (5.8)	0.49 (0.47–0.51)	8/29 (27.6)	3.10 (1.34–6.11)	6.45 (3.22–12.91)	0.77 (0.57–1.05)
Three-year follow-up						
Primary analysis						
Hyperglycemia	5396/55,954 (9.6)	0.28 (0.27–0.29)	1064/5851 (18.2)	0.58 (0.54–0.61)	2.07 (1.94–2.21)	1.32 (1.21–1.44)
Hypoglycemia	6290/60,353 (10.4)	0.31 (0.30–0.31)	170/1452 (11.7)	0.35 (0.30–0.41)	1.14 (0.98–1.32)	0.88 (0.73–1.05)
Both	6426/61,660 (10.4)	0.31 (0.30–0.31)	34/145 (23.4)	0.77 (0.54–1.08)	2.34 (1.67–3.28)	3.89 (1.02–14.73)
Subgroup analysis						
With diabetes						
Hyperglycemia	1671/18,452 (9.1)	0.26 (0.25–0.28)	554/3133 (17.7)	0.55 (0.51–0.60)	2.07 (1.88–2.27)	1.40 (1.25–1.56)
Hypoglycemia	2132/20,759 (10.3)	0.30 (0.29–0.31)	93/826 (11.3)	0.33 (0.27–0.41)	1.12 (0.91–1.37)	0.99 (0.78–1.27)
Both	2199/21,469 (10.2)	0.30 (0.29–0.31)	26/116 (22.4)	0.72 (0.47–1.05)	2.23 (1.51–3.28)	1.79 (0.96–3.34)
Without diabetes						
Hyperglycemia	3725/37,502 (9.9)	0.29 (0.28–0.30)	510/2718 (18.8)	0.61 (0.55–0.66)	2.19 (2.00–2.41)	1.24 (1.07–1.45)
Hypoglycemia	4158/39,594 (10.5)	0.31 (0.30–0.32)	77/626 (12.3)	0.37 (0.30–0.47)	1.19 (0.95–1.49)	0.95 (0.49–1.87)
Both	4227/40,191 (10.5)	0.31 (0.30–0.32)	8/29 (27.6)	1.04 (0.45–2.05)	3.23 (1.61–6.46)	8.07 (2.10–31.05)

HR = hazard ratio; CI = confidence interval.

## Data Availability

The raw data supporting the conclusions of this article will be made available by the authors on request.

## Supplementary Materials

The following supporting information can be downloaded at: https://www.mdpi.com/article/10.3390/jcm13040932/s1, Figure S1. Cumulative incidence of one-year mortality of the exposed group compared with the unexposed group; Figure S2. Cumulative incidence of three-year mortality of the exposed group compared with the unexposed group; Table S1. Subgroup results among patients without psychiatric disorders; Table S2. Baseline characteristics according to presence of preoperative hyperglycemia before and after inverse probability of weighting (IPW) adjustment; Table S3. Baseline characteristics according to presence of preoperative hypoglycemia before and after inverse probability of weighting (IPW) adjustment; Table S4. Baseline characteristics according to presence of both hyper- and hypoglycemia before and after inverse probability of weighting (IPW) adjustment.
