# Supplementary material for: Association between Preoperative Glucose Dysregulation and Delirium after Non-Cardiac Surgery
Source: J Clin Med. 2024 Feb 6;13(4):932. doi: 10.3390/jcm13040932 (PMC10889204; doi:10.3390/jcm13040932)
Supplement: Supplementary file 1 [file jcm-13-00932-s001.zip › jcm-2796593-supplementary.pdf]

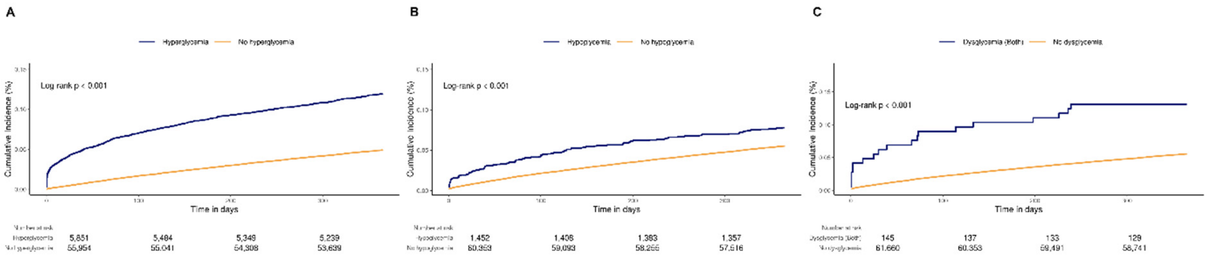

Figure S1. Cumulative incidence of one-year mortality of the exposed group compared with the unexposed group.

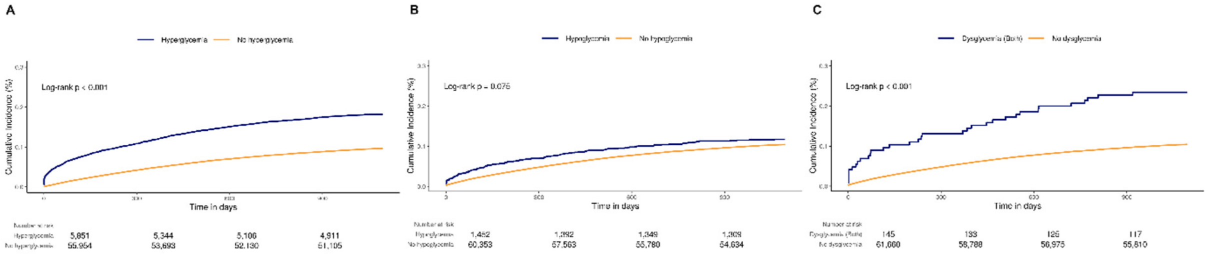

Figure S2. Cumulative incidence of three-year mortality of the exposed group compared with the unexposed group.

Table S1. Subgroup results among patients without psychiatric disorders.

Outcome	Unexposed group		Exposed group			
	No. with outcome/total<br>No. (%)	Incidence rate per 100 person-months (95% CI)	No. with outcome/total<br>No. (%)	Incidence rate per 100 person-months (95% CI)	Crude HR (95% CI)	IPTW-adjusted HR (95% CI)
Delirium						
Hyperglycemia	1352/53508 (2.5)	2.53 (2.51-2.54)	341/5426 (6.3)	6.30 (6.23-6.37)	2.54 (2.25-2.86)	1.37 (1.18-1.60)
Hypoglycemia	1616/57560 (2.8)	2.81 (2.80-2.82)	77/1374 (5.6)	5.62 (5.49-5.74)	2.03 (1.61-2.55)	1.41 (1.08-1.84)
Both	1677/58797 (2.9)	2.85 (2.84-2.87)	16/137 (11.7)	11.77 (11.20-12.36)	4.32 (2.64-7.06)	1.83 (0.78-4.32)
Mortality (One-year follow-up)						
Hyperglycemia	2586/53508 (4.8)	0.47 (0.46-0.47)	661/5426 (12.2)	1.29 (1.28-1.30)	2.74 (2.51-2.98)	1.34 (1.20-1.51)
Hypoglycemia	3138/57560 (5.5)	0.53 (0.53-0.53)	109/1374 (7.9)	0.79 (0.77-0.80)	1.48 (1.22-1.79)	1.04 (0.83-1.30)
Both	3228/58797 (5.5)	0.53 (0.53-0.54)	19/137 (13.9)	1.44 (1.38-1.51)	2.69 (1.71-4.22)	1.11 (0.51-2.44)
Mortality (Three-year follow-up)						

Hyperglycemia	5108/53508 (9.5)	0.37 (0.37-0.37)	994/5426 (18.3)	0.80 (0.79-0.80)	2.12 (1.98-2.27)	1.33 (1.22-1.45)
Hypoglycemia	5939/57560 (10.3)	0.41 (0.41-0.41)	163/1374 (11.9)	0.47 (0.47-0.48)	1.17 (1.00-1.36)	0.89 (0.74-1.07)
Both	6070/58797 (10.3)	0.41 (0.41-0.41)	32/137 (23.4)	0.96 (0.93-1.00)	2.38 (1.68-3.36)	1.24 (0.69-2.24)

Table S2. Baseline characteristics according to presence of preoperative hyperglycemia before and after inverse probability of weighting (IPW) adjustment.

	Unexposed	Exposed	ASD	
	(N=55,954)	(N=5,851)	Before IPW	After IPW
Male	29375 (52.5)	3430 (58.6)	12.3	2.3
Age	57.4 (±14.7)	60.9 (±13.3)	24.9	3.8
Body mass index	24.4 (±3.7)	24.3 (±2.2)	3.2	0.9
Psychiatric disorder, any	2446 (4.4)	425 (7.3)	12.4	0.6
Mood disorder	1085 (1.9)	162 (2.8)	5.5	2.1
Schizophrenia	69 (0.1)	15 (0.3)	3.1	0.2
Alcoholic use disorder	64 (0.1)	11 (0.2)	1.9	0.8
Other substance abuse	14 (0.0)	4 (0.1)	2	<0.1
Sleep disorder	831 (1.5)	118 (2.0)	4.1	0.3
Personality disorder	18 (0.0)	4 (0.1)	1.6	0.7
Current alcohol	10461 (18.7)	946 (16.2)	6.7	1.1
Current smoking	4891 (8.7)	498 (8.5)	0.8	1.2
Previous disease				
Hypertension	21242 (38.0)	2679 (45.8)	15.9	2.9
Diabetes	18452 (33.0)	3133 (53.5)	42.4	1.8
Chronic kidney disease	1954 (3.5)	409 (7.0)	15.7	2.1
Dialysis	634 (1.1)	161 (2.8)	11.7	1.9
Stroke	2028 (3.6)	406 (6.9)	14.9	1.3
Coronary artery disease	1988 (3.6)	280 (4.8)	6.2	1
Heart failure	377 (0.7)	62 (1.1)	4.2	1.6
Arrhythmia	1393 (2.5)	195 (3.3)	5	1
Peripheral artery disease	333 (0.6)	69 (1.2)	6.2	0.5
Aortic disease	362 (0.6)	38 (0.6)	<0.1	2.3
Valvular heart disease	125 (0.2)	20 (0.3)	2.2	0.1
Chronic obstructive pulmonary disease	1409 (2.5)	148 (2.5)	0.1	0.4
Preoperative blood laboratory tests				
Hemoglobin, g/dl	13.1 (±1.9)	12.7 (±2.2)	22.1	4.1
Creatinine, mg/dL	1.0 (±1.3)	1.2 (±1.5)	14	2.8
Preoperative electrolytes				
Hyponatremia	2909 (5.2)	789 (13.5)	28.8	3.1

Hypernatremia	500 (0.9)	119 (2.0)	9.5	0.6
Hypokalemia	1147 (2.0)	288 (4.9)	15.7	1
Hyperkalemia	702 (1.3)	146 (2.5)	7.2	1
Hypophosphatemia	1773 (3.2)	405 (6.9)	17.2	1.9
Hyperphosphatemia	2250 (4.0)	310 (5.3)	6.1	1
Hypochloremia	1751 (3.1)	506 (8.6)	23.6	3.2
Hyperchloremia	9457 (16.9)	942 (16.1)	2.2	1.9
Operative variables				
General anesthesia	50234 (89.8)	5186 (88.6)	3.7	2.8
Emergency operation	6702 (12.0)	2030 (34.7)	55.8	0.1
Operation duration, min	170.2 (±124.9)	149.8 (±115.7)	14	2.9
Intraoperative transfusion	4243 (7.6)	696 (11.9)	14.6	3.4
Intraoperative inotropics infusion	7736 (13.9)	619 (10.6)	10.1	0.4
Surgical risk				
Mild	11741 (21.0)	1190 (20.3)	1.6	4.9
Intermediate	36450 (65.1)	4042 (69.1)	8.4	3.4
High	7763 (13.9)	619 (10.6)	10.1	1.1
Surgery types			31.4	
Neuroendocrine	1244 (2.2)	81 (1.4)		
Lung	2759 (4.9)	533 (9.1)		
Head & Neck	11956 (21.4)	1410 (24.1)		
Breast	1274 (2.3)	128 (2.2)		
Stomach	2694 (4.8)	239 (4.1)		
Hepatobiliary	8371 (15.0)	859 (14.7)		
Colorectal	4373 (7.8)	550 (9.4)		
Urology	5836 (10.4)	390 (6.7)		
Gynecology	3755 (6.7)	137 (2.3)		
Bone & Skin etc	13692 (24.5)	1524 (26.0)		

Table S3. Baseline characteristics according to presence of preoperative hypoglycemia before and after inverse probability of weighting (IPW) adjustment.

	Unexposed (N=60,353)	Exposed (N=1,452)	ASD Before IPW	ASD After IPW
Male	32099 (53.2)	706 (48.6)	9.1	1.3
Age	57.8 (±14.6)	58.0 (±14.7)	1.6	1.2
Body mass index	24.4 (±3.7)	23.9 (±3.8)	14.5	2.7
Psychiatric disorder, any	2793 (4.6)	78 (5.4)	3.4	0.4
Mood disorder	1217 (2.0)	30 (2.1)	0.4	0.3
Schizophrenia	83 (0.1)	1 (0.1)	2.1	0.3
Alcoholic use disorder	72 (0.1)	3 (0.2)	2.2	0.4
Other substance abuse	18 (0.0)	0	2.4	2.4

Sleep disorder	923 (1.5)	26 (1.8)	2	0.3
Personality disorder	22 (0.0)	0	2.7	2.7
Current alcohol	11194 (18.5)	213 (14.7)	10.4	1.2
Current smoking	5262 (8.7)	127 (8.7)	0.1	0.2
Previous disease				
Hypertension	23312 (38.6)	609 (41.9)	6.8	0.6
Diabetes	20759 (34.4)	826 (56.9)	46.4	0.8
Chronic kidney disease	2222 (3.7)	141 (9.7)	24.3	3.7
Dialysis	703 (1.2)	92 (6.3)	27.5	1
Stroke	2370 (3.9)	64 (4.4)	2.4	0.7
Coronary artery disease	2199 (3.6)	69 (4.8)	5.5	1.3
Heart failure	416 (0.7)	23 (1.6)	8.4	0.3
Arrhythmia	1546 (2.6)	42 (2.9)	2	0.5
Peripheral artery disease	391 (0.6)	11 (0.8)	1.3	2.6
Aortic disease	392 (0.6)	8 (0.6)	1.3	1.4
Valvular heart disease	144 (0.2)	1 (0.1)	4.3	5
Chronic obstructive pulmonary disease	1527 (2.5)	30 (2.1)	3.1	2.4
Preoperative blood laboratory tests				
Hemoglobin, g/dl	13.1 (±2.0)	12.3 (±1.9)	38.9	6.1
Creatinine, mg/dL	1.0 (±1.3)	1.6 (±2.3)	29.5	3.5
Preoperative electrolytes				
Hyponatremia	3562 (5.9)	136 (9.4)	13.1	1.3
Hypernatremia	602 (1.0)	17 (1.2)	1.7	2.3
Hypokalemia	1397 (2.3)	38 (2.6)	1.9	0.4
Hyperkalemia	765 (1.3)	83 (5.7)	24.4	0.2
Hypophosphatemia	2118 (3.5)	60 (4.1)	3.2	3.6
Hyperphosphatemia	2431 (4.0)	129 (8.9)	19.9	1.7
Hypochloremia	2165 (3.6)	92 (6.3)	12.7	2.5
Hyperchloremia	10118 (16.8)	281 (19.4)	6.7	0.3
Operative variables				
General anesthesia	54161 (89.7)	1259 (86.7)	9.4	0.4
Emergency operation	8494 (14.1)	238 (16.4)	6.5	5.5
Operation duration, min	168.3 (±124.4)	168.5 (±114.0)	0.2	<0.1
Intraoperative transfusion	4747 (7.9)	192 (13.2)	17.5	0.1
Intraoperative inotropics infusion	8706 (14.4)	317 (21.8)	19.3	0.9
Surgical risk				
Mild	12677 (21.0)	254 (17.5)	8.9	2.9
Intermediate	39557 (65.5)	935 (64.4)	2.4	3.7
High	8119 (13.5)	263 (18.1)	12.8	1.8
Surgery types			25.7	
Neuroendocrine	1297 (2.1)	28 (1.9)		

Lung	3242 (5.4)	50 (3.4)		
Head & Neck	13102 (21.7)	264 (18.2)		
Breast	1385 (2.3)	17 (1.2)		
Stomach	2866 (4.7)	67 (4.6)		
Hepatobiliary	8989 (14.9)	241 (16.6)		
Colorectal	4811 (8.0)	112 (7.7)		
Urology	6032 (10.0)	194 (13.4)		
Gynecology	3731 (6.2)	161 (11.1)		
Bone & Skin etc	14898 (24.7)	318 (21.9)		

Table S4. Baseline characteristics according to presence of both hyper- and hypoglycemia before and after inverse probability of weighting (IPW) adjustment.

	Unexposed	Exposed	ASD	
	(N=61,660)	(N=145)	Before IPW	After IPW
Male	32722 (53.1)	83 (57.2)	8.4	2.8
Age	57.8 (±14.6)	61.6 (±12.8)	28.1	7.7
Body mass index	24.4 (±3.7)	22.6 (±3.4)	49.4	4
Psychiatric disorder, any	2863 (4.6)	8 (5.5)	4	4.8
Mood disorder	1246 (2.0)	1 (0.7)	11.5	8.9
Schizophrenia	84 (0.1)	0	5.2	5.2
Alcoholic use disorder	75 (0.1)	0	4.9	4.9
Other substance abuse	18 (0.0)	0	2.4	2.4
Sleep disorder	947 (1.5)	2 (1.4)	1.3	3.6
Personality disorder	22 (0.0(	0	2.7	2.7
Current alcohol	11389 (18.5)	18 (12.4)	16.8	9.9
Current smoking	5380 (8.7)	9 (6.2)	9.6	8.9
Previous disease				
Hypertension	23847 (38.7)	74 (51.0)	25	3.6
Diabetes	21469 (34.8)	116 (80.0)	>99	6.9
Chronic kidney disease	2336 (3.8)	27 (18.6)	48.4	7.3
Dialysis	781 (1.3)	14 (9.7)	37.6	1.1
Stroke	2421 (3.9)	13 (9.0)	20.6	7.5
Coronary artery disease	2261 (3.7)	7 (4.8)	5.8	8.2
Heart failure	435 (0.7)	4 (2.8)	15.8	2.7
Arrhythmia	1579 (2.6)	9 (6.2)	17.9	5.1
Peripheral artery disease	399 (0.6)	3 (2.1)	12.3	2.2
Aortic disease	400 (0.6)	0	11.4	9.5
Valvular heart disease	145 (0.2)	0	6.9	6.9
Chronic obstructive pulmonary disease	1553 (2.5)	4 (2.8)	1.5	6.1
Preoperative blood laboratory tests				
Hemoglobin, g/dl	13.1 (±2.0)	12.0 (±2.0)	56	9.8

Creatinine, mg/dL	1.0 (±1.3)	2.0 (±2.4)	49.1	0.6
Preoperative electrolytes				
Hyponatremia	3678 (6.0)	20 (13.8)	26.5	10.3
Hypernatremia	616 (1.0)	3 (2.1)	8.7	3.7
Hypokalemia	1431 (2.3)	4 (2.8)	2.8	0.6
Hyperkalemia	834 (1.4)	14 (9.7)	37	5.4
Hypophosphatemia	2168 (3.5)	10 (6.9)	15.3	10.2
Hyperphosphatemia	2542 (4.1)	18 (12.4)	30.5	4.8
Hypochloremia	2241 (3.6)	16 (11.0)	28.7	9.2
Hyperchloremia	10371 (16.8)	28 (19.3)	6.5	7.3
Operative variables				
General anesthesia	55295 (89.7)	125 (86.2)	10.7	10.4
Emergency operation	8700 (14.1)	32 (22.1)	20.8	10.2
Operation duration, min	168.3 (±124.2)	157.4 (±117.6)	9	6.5
Intraoperative transfusion	4917 (8.0)	22 (15.2)	22.6	5.9
Intraoperative inotropics infusion	8979 (14.6)	44 (30.3)	38.5	9.7
Surgical risk				
Mild	12894 (20.9)	37 (25.5)	10.9	5.8
Intermediate	40409 (65.5)	83 (57.2)	17.1	7.4
High	8357 (13.6)	25 (17.2)	10.2	3.5
Surgery types			36.6	
Neuroendocrine	1322 (2.1)	3 (2.1)		
Lung	3281 (5.3)	11 (7.6)		
Head & Neck	13344 (21.6)	22 (15.2)		
Breast	1397 (2.3)	5 (3.4)		
Stomach	2925 (4.7)	8 (5.5)		
Hepatobiliary	9209(14.9)	21 (14.5)		
Colorectal	4919 (8.0)	14 (9.7)		
Urology	6203 (10.1)	23 (15.9)		
Gynecology	3890 (6.3)	2 (1.4)		
Bone & Skin etc	15180 (24.6)	36 (24.8)		
